# Blockage of Autophagy for Cancer Therapy: A Comprehensive Review

**DOI:** 10.3390/ijms25137459

**Published:** 2024-07-07

**Authors:** Ahmed Mostafa Ibrahim Abdelrahman Hassan, Yuxin Zhao, Xiuping Chen, Chengwei He

**Affiliations:** 1 State Key Laboratory of Quality Research in Chinese Medicine, Institute of Chinese Medical Sciences, University of Macau, Taipa, Macao SAR 999078, Chinaxpchen@um.edu.mo (X.C.); 2 Department of Pharmaceutical Science, Faculty of Health Sciences, University of Macau, Taipa, Macao SAR 999078, China

**Keywords:** autophagy inhibitors, cancer therapy, clinical trials

## Abstract

The incidence and mortality of cancer are increasing, making it a leading cause of death worldwide. Conventional treatments such as surgery, radiotherapy, and chemotherapy face significant limitations due to therapeutic resistance. Autophagy, a cellular self-degradation mechanism, plays a crucial role in cancer development, drug resistance, and treatment. This review investigates the potential of autophagy inhibition as a therapeutic strategy for cancer. A systematic search was conducted on Embase, PubMed, and Google Scholar databases from 1967 to 2024 to identify studies on autophagy inhibitors and their mechanisms in cancer therapy. The review includes original articles utilizing in vitro and in vivo experimental methods, literature reviews, and clinical trials. Key terms used were “Autophagy”, “Inhibitors”, “Molecular mechanism”, “Cancer therapy”, and “Clinical trials”. Autophagy inhibitors such as chloroquine (CQ) and hydroxychloroquine (HCQ) have shown promise in preclinical studies by inhibiting lysosomal acidification and preventing autophagosome degradation. Other inhibitors like wortmannin and SAR405 target specific components of the autophagy pathway. Combining these inhibitors with chemotherapy has demonstrated enhanced efficacy, making cancer cells more susceptible to cytotoxic agents. Clinical trials involving CQ and HCQ have shown encouraging results, although further investigation is needed to optimize their use in cancer therapy. Autophagy exhibits a dual role in cancer, functioning as both a survival mechanism and a cell death pathway. Targeting autophagy presents a viable strategy for cancer therapy, particularly when integrated with existing treatments. However, the complexity of autophagy regulation and the potential side effects necessitate further research to develop precise and context-specific therapeutic approaches.

## 1. Introduction

In recent years, the overall incidence and mortality of cancer have continued to increase. Cancer has become one of the leading causes of death, with a reported worldwide mortality rate of nearly 10 million in 2020 [1,2,3]. Currently, surgical interventions, radiotherapy, and chemotherapy are the main treatments for cancers [4,5,6]. However, survival rates in metastatic cancers remain unsatisfactory, attributed to the complex nature of treating cancers and therapeutic resistance [7]. Additionally, many cellular adaptations may affect drug efficacy, one of which is autophagy, the focus of this review. 

Autophagy plays a crucial role in the occurrence, drug resistance, and treatment of cancer [8]. It is a multi-step self-degradation mechanism in a wide range of cells, acting as a recycling system within living cells by degrading misfolded proteins and damaged or aging organelles through the lysosomes [9,10]. It plays a vital role in cell proliferation, apoptosis, migration, and the invasion of tumors [11,12]. Moreover, autophagy is usually maintained at basal levels in normal conditions to maintain homeostasis and facilitate adaptation under stress as a cytoprotective process. When cells encounter extrinsic stresses, including reactive oxygen species (ROS), long-lived proteins, infection, and damaged mitochondria, autophagy transports these components to lysosomes for degradation and recycling [13]. 

The term “autophagy” was coined by Christian de Duve in 1963, referring to “self-eating” and describing the mechanism by which cells break down their components to maintain cellular homeostasis [14]. Since then, there has been increasing research focusing on explaining the relationship between autophagy and various diseases. Dysregulated autophagy has been implicated in a wide range of pathologies, and its modulation by targeting regulatory factors can potentially impact disease progression. In malignancies, for example, the role of autophagy varies among tumor-suppressive, tumor-promoting, or neutral effects depending on the context, highlighting its complexity and the reasons for chemoresistance [15,16,17]. Consequently, many researchers are investigating the potential of autophagy inhibitors as a treatment strategy for various types of cancer, and for understanding the complex interactions in patients [18,19]. By inhibiting autophagy, the deprivation of necessary nutrients and energy sources in cancer cells will cause cell apoptosis and necrosis. This process can be achieved by autophagy inhibitors (either natural or synthetic), which have shown promising results in preclinical studies, with many agents progressing to clinical trials as standalone treatments or in combination with standard-of-care therapeutics. Notably, most current autophagy inhibitors are repurposed agents, previously used for other diseases, such as chloroquine (CQ) and hydroxychloroquine (HCQ) in malaria, and they have progressed to the clinic faster than early-stage development drugs. However, their clinical efficacy is still under investigation [20].

## 2. The General Biology of Autophagy

As previously discussed, autophagy is a highly conserved cellular process responsible for the degradation of damaged intracellular components and the production of recyclable molecules such as glucose, ATP, amino acids, and fatty acids. This process plays a crucial and indispensable role in cellular degradation, functioning in conjunction with the highly specific proteasomal degradation pathway, known as the ubiquitin–proteasome system [21,22]. Furthermore, autophagy is essential for maintaining cellular homeostasis and can be categorized into three distinct types: chaperone-mediated autophagy, microautophagy, and macroautophagy. These different types of autophagy dispose of cytoplasmic components by transporting them into lysosomes [23,24]. Despite having a common goal, the mechanisms of cytoplasmic sequestration and the pathways used to transport targeted proteins differ among these types. When considering chaperone-mediated autophagy, it specifically requires the presence of certain proteins such as heat shock protein 70 or lysosome-associated membrane protein 2A for lysosomal degradation [25].

Additionally, autophagy can be divided into microautophagy, which captures cargo by utilizing membrane-bound structures and directly enters the lysosome through the invagination of the lysosome membrane without forming an autophagosome [26]; and macroautophagy, which can be divided into five distinct stages: initiation, nucleation, elongation, maturation, fusion, and degradation, which are tightly regulated by a complex network to ensure the sequential advancement of this vital cellular process [11,21]

### Molecular Mechanisms and Phases of Autophagy

**Figure 1:**
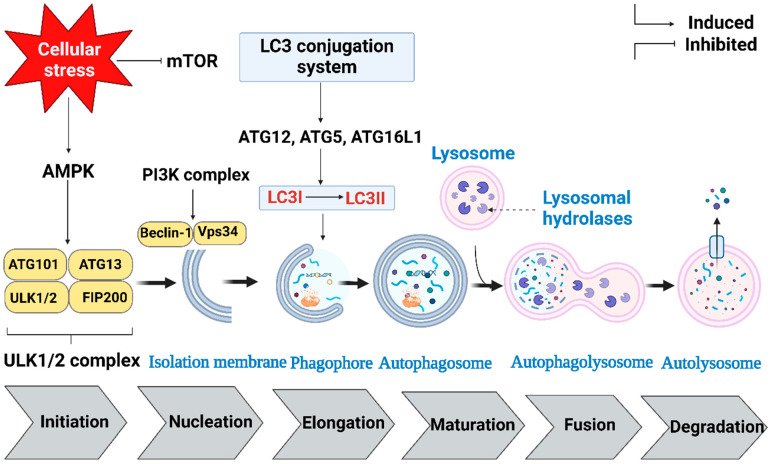
The mechanism of autophagy.

As shown in Figure 1, the regulation of autophagy at the molecular level primarily occurs through the canonical pathway, which integrates key components like Unc-51-like kinase 1 (ULK1), the phosphoinositide 3-kinase (PI3K) complex, the microtubule-linked protein 1-light chain 3 (LC3) conjugation system, and lysosomal hydrolases. This pathway involves a complex interplay of signaling pathways and proteins that govern the initiation and progression of autophagy [20].

The initiation phase of autophagy begins with the activation of autophagy machinery in response to specific signals, such as cellular stress [27]. This intricate process is orchestrated by a network of signaling pathways, involving the ULK1 protein [20,28,29].

Following initiation, the process advances to the nucleation phase. This phase is characterized by the formation of a double-membrane structure called the isolation membrane, mediated by the class III PI3K complex, specifically targeting VPS34 [30]. The isolation membrane elongates to form a structure known as the phagophore. The elongation and maturation of the phagophore are facilitated by the LC3 conjugation system, which involves autophagy-related genes (ATG), particularly the ATG12–ATG5-ATG16L1 complex. This complex plays a crucial role in promoting LC3 lipidation and autophagosomal membrane formation, ultimately leading to the creation of a completed autophagosome [31].

The final stages of autophagy involve fusion and degradation. During the fusion step, the autophagosome merges with a lysosome to form an autolysosome. Once the autolysosome is formed, lysosomal hydrolases acidify its interior, facilitating the degradation of the autophagic cargo [32].

Consequently, a comprehensive understanding of the molecular mechanisms and phases of autophagy is crucial for developing effective inhibitors that impact this vital cellular process. Surprisingly, many inhibitors targeting different stages of autophagy have been identified and studied for their therapeutic potential. For instance, ULK1 inhibitors disrupt the initiation phase [33], while class III PI3K inhibitors, targeting VPS34, impede the nucleation phase [34,35,36]. Autophagosome maturation can be inhibited by agents that interfering with ATG [37]. Additionally, lysosomotropic agents prevent the autophagosomes’ fusion with lysosomes, thereby preventing the formation of autolysosomes and subsequent cargo degradation [38,39].

## 3. The Regulation of Autophagy

As autophagy progresses through maturation and degradation, the mechanistic target of rapamycin kinase (mTOR) and AMP-activated kinase (AMPK) signaling pathways play crucial roles as regulators. These nutrient sensors establish complex connections with the autophagy machinery, influencing the dynamics and outcomes of the process. Both mTOR and AMPK are extensively studied and well-understood as the central regulators of autophagy [40,41]. Their coordinated activation and inhibition effectively control the sequestration, transport, fusion, and degradation of cargo in the final stage of autophagy, providing critical insight into the precise coordination required for autophagy completion. While other pathways and molecules are implicated in autophagy regulation, mTOR and AMPK remain the focus of extensive research and comprehension.

### 3.1. Mammalian Target of Rapamycin (mTOR)

The mTOR pathway functions as the central negative regulator of autophagy [42]. It is a critical serine/threonine kinase that regulates cell growth, metabolism, and autophagy [43]. The activation of mTOR occurs under conditions such nutrient abundance, environmental/cellular stress-free conditions (hypoxia, heat shock, osmotic stress, ROS, DNA damage, and endoplasmic reticulum stress), and sufficient growth factors [41,43,44]. In contrast, nutrient scarcity leads to mTOR inhibition, and initiates autophagy activation [41].

### 3.2. AMP-Activated Protein Kinase (AMPK)

The AMPK pathway plays a critical role in maintaining ATP levels within the cell and responds to various cellular stresses, such as hypoxia, nutrient deprivation, and mitochondrial stress [45,46]. It triggers autophagy by influencing a variety of proteins, including ULK1 and Beclin-1 [47].

In fact, the role of AMPK in autophagy regulation has been well studied. The studies found that AMPK is required for autophagy induction in response to glucose deprivation and inhibiting its function results in disrupted autophagy flux and the accumulation of autophagosomes. In summary, under conditions of glucose deprivation, AMPK promotes the activation of ULK1 and Beclin-1 via phosphorylation at specific sites, highlighting its pivotal role as a regulator of autophagy initiation [44,48,49,50].

## 4. Bipolar Nature of Autophagy in Cancer

Many studies have been focused on elucidating the relationship between autophagy and the progression of cancer. The impact of autophagy on the fate of tumor cells is contingent upon factors such as the type of cancer, its stage, and the genetic characteristics involved [51]. The multifaceted role of autophagy in cancer therapy exhibits context-dependent complexity, manifesting in two distinct responses to anticancer drugs [52]. One response is the cytotoxic function known as autophagic cell death, which prevents mutations [53,54]. The other response is the cytoprotective function, which leads to drug resistance. As a result, this mechanism poses a significant clinical challenge for achieving successful cancer treatment and results in poor patient prognosis [55].

### 4.1. Tumor-Suppressive Role of Autophagy

Autophagic cell death, also known as type II programmed cell death, is a non-apoptotic process induced by an anticancer treatment that promotes the autophagy of cancer cells and culminates in cell death due to overactivated autophagy [56,57,58,59]. Moreover, activating autophagy-related signaling pathways may promote the degradation of potentially oncogenic molecules, thereby contributing to the defense against tumor invasion, angiogenesis, and migration [60,61,62].

Furthermore, autophagic cell death is closely associated with the regulation of pivotal proteins such as Beclin-1, which plays a vital role in tumor suppression. Under normal conditions and early stages of cancer, Beclin-1 safeguards cells against adverse stimuli and stress, thus maintaining cellular function and integrity. Additionally, it counteracts the detrimental effects of ROS, which are implicated in triggering mutations and DNA damage associated with cancer progression. By triggering autophagy in response to increased ROS levels, Beclin-1 facilitates the removal of damaged cellular components, preserving genomic stability and mitigating oxidative stress [63,64]. Nonetheless, disturbances in the regulation of Beclin-1 may compromise autophagy, potentially leading to genome instability and tumorigenesis [65]. Overall, autophagy prevents tumor initiation, and its dysfunction can contribute to tumorigenesis.

### 4.2. Tumor-Promoting Role of Autophagy

Given that autophagy serves as a survival mechanism and stress response, it also contributes to the endurance of established tumor cells under various stress conditions. Furthermore, autophagy enhances stress tolerance in developed tumors and provides an alternative pathway for cancer cells to sustain their energy requirements and procure vital nutrients [16,17]. This protection is achieved through the breakdown of macromolecules and the recycling of the essential building blocks that fuel the metabolism of tumor cells. Thus, it nullifies the effect of drugs. Therefore, the upregulation of autophagy can offer tumor cells a competitive advantage over normal cells, fostering aggressiveness and resistance to cancer therapy [66].

## 5. The Rationale for Targeting Autophagy in Cancer Therapy

Autophagy modulation has gained significant attention as a promising strategy for cancer therapy. Based on the above information, autophagy plays a dual role in cancer, working as both a cytoprotective and a cytotoxic factor for cancer cells. Hence, autophagy can function as a tumor promoter or suppressor. This complexity makes it a potential target for cancer therapy using autophagy inhibitors. In detail, cancer cells rely on autophagy for survival in adverse conditions like nutrient deprivation and hypoxia, making it an attractive therapeutic target due to its high energy demands [67].

Moreover, targeting autophagy can enhance the efficacy of existing chemotherapy drugs. When autophagy is inhibited, cancer cells become more susceptible to the cytotoxic effects of anticancer agents, indicating a potential method for improving treatment outcomes in various cancers, including breast, ovarian, and melanoma [68,69,70,71]. As a result, autophagy presents a promising approach to cancer therapy [72,73].

However, the debate on whether to activate or inhibit autophagy remains intense due to the complexity of autophagy physiology, and no consensus has been reached. Drug targets have been identified at almost every step of autophagy, from initiation to vesicle nucleation and maturation, vesicle fusion, and lysosomal degradation. Understanding these regulatory pathways could help develop new cancer treatment strategies [74]. Further research is necessary to explore precise and context-specific strategies for effectively utilizing autophagy inhibitors in cancer treatment.

However, drug resistance poses a significant challenge in cancer therapy, necessitating novel approaches. Increasing research has demonstrated that drug resistance in cancer therapy can be overcome by pharmacologically inhibiting autophagy using inhibitors targeting critical components within the autophagy pathway, or through genomic interference against autophagic genes such as small interfering RNA (siRNA), targeting *ATG3*, *ATG5*, and *ATG7* [75]. Fortunately, blocking autophagy pharmacologically or genetically has shown promising results in inducing tumor regression in genetic mouse models and pancreatic cancer xenografts, highlighting them as promising therapies for targeting autophagy [76].

Up to now, autophagic inhibitors can be categorized into three classes. The first category comprises Class III PI3K inhibitors that target the VPS34 enzyme involved in autophagy and vesicle dynamics. The other two main types of PI3K inhibitors are Class I inhibitors, which intercept oncogenic PI3Ks, and Class II inhibitors, which regulate membrane trafficking. Collectively, these three PI3K pathways are favored targets for anticancer drug development due to their impact on crucial cellular processes like signaling and cytoskeletal dynamics, resulting in augmented activity of chemotherapies (e.g., taxol and doxorubicin) [34,35,36]. The second category consists of lysosomotropic agents that block autophagic progression by repressing lysosomal acidification [38,39]. The third category includes autophagosome–lysosome fusion inhibitors, which impede the fusion of autophagosomes with lysosomes during the maturation stage, consequently halting the autophagy process [77].

Within the spectrum of autophagy inhibitors, it is worth noting that combining natural products with chemotherapy is a prevalent approach to enhance the anticancer effects while mitigating the dose-dependent adverse effects of cancer treatment. Considering natural products, one of the most prominent components used extensively in healthcare is traditional Chinese medicine (TCM), especially herbal medicine. Recent research findings investigate the potential novel applications of TCMs via the regulation of autophagy, emphasizing their therapeutic effect in cancer treatment [78]. For instance, oxymatrine targets the PI3K pathway, which plays a crucial role in cell growth and survival [79]. Additionally, toosendanin (TSN) and berbamine function as lysosomotropic agents, disrupting the lysosomal activity essential for autophagy [80,81]. This multi-targeted approach of TCM highlights its potential as a valuable compound in cancer therapy.

Furthermore, contemporary scientific investigations have sparked increasing interest in exploring the impact of ULK inhibitors on cellular processes, including autophagy. These inhibitors form complexes with ULK1 regulatory units and operate by impeding kinases in clinical settings, making ULK1 an attractive candidate for autophagy inhibition [33]. Apart from ULK inhibitors, considerable research endeavors have concentrated on comprehending and utilizing the potential of tyrosine kinase and proteasome inhibitors for autophagy suppression as a prospective therapeutic strategy for cancer therapy.

In addition to the main classes mentioned, there are other subordinate classes, such as ATG inhibitors. By utilizing genomic interference techniques, for example, RNA interference (RNAi) and CRISPR, these inhibitors can disrupt *ATG3*, *ATG5*, *ATG7*, and *BECN1*, impairing the complex formation that is involved in the elongation of phagophores, thereby modulating autophagy [37].

## 6. The Classes of Pharmacological Inhibitors Targeting Key Components of Autophagy

**Figure 2:**
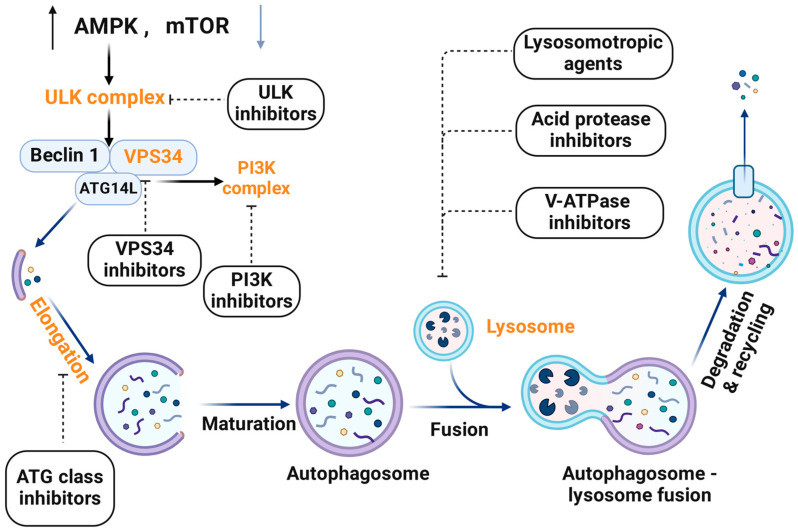
Inhibitors targeting key components of autophagy at each stage.

Given the important roles of autophagy in tumorigenesis and cancer therapy, the inhibition of autophagy is an attractive strategy to enhance the anticancer activity of conventional therapeutic drugs. An increasing number of autophagy inhibitors have been identified. Autophagy can be inhibited at both early (initiation) and late (autophagosome–lysosome fusion and cargo degradation) stages of autophagic flux (Figure 2). The preclinical studies as well as the mechanisms of the major autophagy inhibitors for anticancer therapy are intensively discussed in this section, while others are summarized in Table 1.

### 6.1. Class III PI3K Inhibitors

PI3Ks are lipid kinases that regulate diverse cellular processes, including proliferation, survival, adhesion, and motility. Among the different classes of PI3Ks, class III holds significant importance in governing autophagy, with VPS34 being a critical constituent in the class III PI3K signaling pathway. The targeted inhibition of VPS34 has led to the development of specific VPS34 inhibitors, which have gained significant attention for their ability to modulate this critical component of PI3K cascades. By disrupting the formation of autophagosomes, VPS34 inhibitors effectively impede the process. Therefore, the linkage between class III PI3K class and autophagy inhibitors supports the elucidation of the intricate mechanisms involved in autophagy regulation, providing valuable insights for manipulating the autophagy pathway. Notable examples of early-stage autophagy inhibitors within this class include the natural products wortmannin [235,236] and viridiol, as well as their respective synthetic compounds, 3-methyladenine (3-MA), LY294002, and SAR405.

#### 6.1.1. Wortmannin

Wortmannin is a metabolite derived from the culture of *Penicillium funiculosum*, which exhibits a range of pharmacological effects such as weak antifungal properties and highly active anti-inflammatory effects [237,238]. It has gained prominence for its role as an autophagy inhibitor, effectively hindering autophagosome formation by blocking the class III PI3K pathway during the early nucleation stage, with IC_50_ values ranging from 10 to 50 nM [239,240,241]. Moreover, during the early nucleation stage of autophagy, wortmannin disrupts autophagosome formation by making an irreversible covalent bond with the class III PI3K, consequently activating the cell cycle and apoptosis, and rendering it a non-competitive inhibitor of the PI3K/AKT pathway [239,240,241,242,243,244,245,246,247]. Furthermore, wortmannin can irreversibly inhibit the serine-specific auto-kinase activity of mTOR [239], which contributes to its anticancer properties. One study by Rao et al. revealed that the combination of wortmannin and doxorubicin, using size-adjustable micelles, effectively suppressed the growth of breast cancer and melanoma cells in mice [241].

Additionally, when combined with cisplatin, wortmannin was found to enhance the effectiveness of chemotherapy in overcoming cisplatin resistance in platinum-resistant ovarian cancer [248]. However, wortmannin is not a suitable cancer chemotherapeutic agent for individual use due to stability and toxicity issues. Because of this, there is intense interest in developing new analogs of wortmannin to improve its drug-like properties.

#### 6.1.2. Viridiol

A dihydric derivative called phytotoxic viridiol, derived from the antifungal compound viridian, complements the actions of wortmannin by preventing autophagosome formation. Its 9-epi-viridiol derivative has shown considerable cytotoxic effects on KB and Hela cells [249,250]. In addition, a semi-synthetic viridian derivative, PX-866, has been investigated for its ability to inhibit PI3K activity, resulting in decreased growth and motility of various human cancer cells. PX-866, an improved wortmannin analog, demonstrates greater potency and sustained inhibition of PI3K signaling compared to wortmannin. Notably, PX-866 effectively suppresses cancer cell motility and growth at low nanomolar concentrations, whereas higher concentrations of wortmannin are required for similar effects. Therefore, based on these findings, Howes et al. have suggested PX-866 to be a promising analog of wortmannin, indicating potential improvements in therapeutic applications and PI3K-targeted therapies [251,252].

#### 6.1.3. 3-Methyladenine (3-MA)

As mentioned earlier, natural products have demonstrated the potential to inhibit class III PI3K-mediated autophagy, while synthetic compounds have garnered attention for their ability to modulate autophagy. Known as a well-established autophagy inhibitor, 3-MA effectively blocks autophagy at both the initiation and maturation stages by disrupting the interaction between class III PI3K and various ATG partners. Initially identified for its autophagy inhibitory effects in rat hepatocytes, 3-MA has gained widespread use in research due to its ability to enhance the therapeutic effects of anticancer drugs. Mechanistically, 3-MA inhibits human VPS34 enzymatic activity by binding to a unique hydrophobic pocket of VPS34, a distinct feature from other related kinases like PI3Kα. Moreover, 3-MA has been shown to enhance the therapeutic effect of 5-fluorouracil (5-FU) in gastric carcinoma cells, as well as increasing the sensitivity of paclitaxel-resistant HeLa cervical cancer cells to paclitaxel [253]. However, the in vivo applications of 3-MA are limited due to its poor solubility at room temperature. To overcome this drawback, Wu et al. developed 29 derivatives that not only have improved solubility but also exhibit enhanced activity and selectivity. Among these derivatives, three compounds stand out as promising candidates: [4-(3-methyl-3H-purin-6-yl) thiomorpholine], [3-methyl-6-(3-methylpiperidin-1-yl)-3H-purine], and [6-(4-(3,4-dichlorophenyl) piperazin-1-yl)-3-methyl-3H-purine]. These compounds were identified based on the observation of autophagosome formation upon autophagy induction, using LC3 as an autophagy marker [254]. The development of these novel derivatives with improved solubility presents new opportunities for utilizing 3-MA in vivo and further advancing therapeutic strategies and autophagy research.

#### 6.1.4. LY294002

LY294002, known as 2-(4-morpholinyl)-8-phenylchromone, stands out as one of the earliest PI3K blockers [235,255]. While it has been proven effective in targeting DNA-dependent protein kinase and mTOR, its impact on class III PI3K, particularly VPS34, remains limited [256]. Notably, studies have revealed the potential of LY294002 to enhance the cytotoxicity of temozolomide in cutaneous melanoma cell lines. In vitro research has demonstrated the ability of LY294002 to enhance temozolomide-induced growth arrest and induce G0/G1 cell cycle arrest in Mel Z and Mel IL cell lines [257]. Additionally, in SCC-25 cell lines, LY294002 has been found to reduce GLUT1 expression and influence BAD phosphorylation [258]. Moreover, LY294002 has also demonstrated synergistic cytotoxicity when combined with the natural compound curcumin in breast cancer cell lines, positioning it as a potential enhancer of drug-induced apoptosis [259].

#### 6.1.5. SAR405

In the realm of synthetic compounds, SAR405 is at the forefront as an autophagy inhibitor with low molecular weight, high activity, and remarkable specificity. It selectively targets VPS34 as an ATP-competitive inhibitor. Furthermore, SAR405 demonstrates a distinctive selectivity on class I and II isoforms of PI3K-PtdIns3K, and on mTOR, enabling it to disrupt vesicle trafficking from late endosomes to lysosomes and block PIK3C3 catalytic activity. The potential of SAR405 is evident in Pasquier’s research, which highlights the prospect of combining it with everolimus. This combination inhibits cell proliferation in renal cancer, emphasizing the significant role of SAR405 in advancing cancer therapy [260].

The previous compounds, except for wortmannin, affect related lipids and protein kinases such as mTOR and DNA-dependent protein kinase. However, these compounds have low bioactivity and bioavailability, which is essential to consider within clinical trials [256].

### 6.2. Lysosomotropic Agents (Repressors of Lysosomal Acidification)

Lysosomotropic agents refer to substances that are taken into lysosomes in vivo or in vitro [261]. Lysosomes are the final cell organelles in the endocytic process, where they break down macromolecules with the help of hydrolytic enzymes. These enzymes are active within an acidic pH range from 4.5 to 5.5 [262,263]. However, lysosomotropic agents hinder lysosomal acidification and inhibit the degradation of autophagosomes, thus blocking autophagy [264]. This activity is achieved by using pharmacological inhibitors, such as the natural product TSN and its synthetic analogues including CQ, HCQ, Lys0569, and Roc-325.

#### 6.2.1. Toosendanin (TSN)

TSN is a TCM extracted from *Melia toosendan* Sieb et Zucc. It inhibits autophagy by increasing lysosomal pH rather than blocking the fusion of autophagosomes and lysosomes. Moreover, its impact was investigated in both in vitro and in vivo on triple-negative breast cancer (TNBC), when combined with the topoisomerase I inhibitor irinotecan to demonstrate its therapeutic effects on TNBC. The findings revealed that TSN hindered SN-38/irinotecan-induced apoptosis in TNBC cells and significantly induced protective autophagy in tumor xenograft models compared to using SN-38/irinotecan alone [80]. This result highlights the capability of TSN to inhibit autophagy.

#### 6.2.2. Chloroquine (CQ) and Hydroxychloroquine (HCQ)

Upon wrapping up the TSN analysis, the focus shifts to the study of CQ and HCQ medications. CQ is an aminoquinoline that has been certified by the FDA as an anti-malarial remedy since its approval on 31 October 1949 [264]. Additionally, CQ and its derivatives, such as HCQ, are used in the treatment of various diseases including rheumatoid arthritis [265,266], malaria [20], HIV [267], systemic lupus [268,269], and more recently, in the treatment of COVID-19 [270]. The primary mechanism of action for both CQ and HCQ involves the inhibition of lysosomal acidification. CQ interferes with autophagy during the later stages by generating acidic vesicular organelles in the cytoplasm [271,272]. When lysosomes are exposed to an acidic environment, they continuously accumulate CQ and HCQ, leading to an increase in lysosomal pH due to their alkaline properties, thus blocking the activity of hydrolytic enzymes. Consequently, lysosomal digestion and autophagy are suppressed because of the damage of cytoplasmic proteins and endoplasmic reticulum stress, ultimately leading to apoptosis [272].

Interestingly, a study has explored the synergistic potential of combining natural products with CQ. For instance, the combination of CQ with a tetrandrine natural compound exhibited significant antitumor activity, indicating the possibility of enhancing therapeutic outcomes through this combination therapy [135]. Additionally, the combined effect of another natural product called resveratrol with HCQ was investigated on an osteosarcoma cell line (MG-63). This study revealed a synergistic effect and further emphasized the potential of combining autophagy inhibitors with natural products for enhanced therapeutic efficacy [134]. Furthermore, medical progress has prompted investigations into the optimal dosage of CQ and HCQ that balances safety and effectiveness in cancer treatment. Karim et al. conducted a phase I study to evaluate the maximum tolerated dose of CQ and HCQ in combination with carboplatin-gemcitabine in advanced solid tumors. It was observed that the maximum tolerated doses of CQ and HCQ are lower when used concomitantly with the previously reported chemotherapeutic regimes due to the myelosuppressive action of carboplatin–gemcitabine [273]. This study highlights the potential of CQ or HCQ to yield synergistic antitumor effects, pointing towards the possibility of the development of novel therapeutic drugs in cancer treatment.

Although CQ and HCQ have been extensively studied for their potential in various medical applications, a new synthetic drug known as Lys05 is now emerging as a promising competitor. Lys05 is a novel lysosomal autophagy inhibitor currently in development. It is a water-soluble salt derived from its parent compound, Lys01. However, Lys05 tends to accumulate within the lysosome, causing deacidification and leading to a more significant autophagy inhibition. In two models of melanoma xenograft and a model of colon cancer xenograft, lower doses of Lys05 blocked early autophagy obviously and showed effects as a single antitumor agent. Moreover, Lys05 has a higher potency than CQ and HCQ, with 10 times more cytotoxic and antitumor activity [136,137].

#### 6.2.3. ROC-325

ROC-325 has been developed by applying a logical medicinal chemistry approach to drug design. This compound leads to autophagosome accumulation and lysosome deacidification at low doses, and it has been administered orally in some in vivo studies using mice. The analysis of tumor samples from mice treated with ROC-325 indicated its ability to inhibit autophagy and decrease tumor cell proliferation [154]. In another in vivo study, ROC-325 was found to enhance the activity of azacitidine (AZA) as an anti-leukemic agent in acute myeloid leukemia by inducing increased LC3B and p62 levels. It increased the anti-leukemic activity of AZA by antagonizing its effects on p62, while also reducing the suppressive effect of AZA on autophagy induction [153].

### 6.3. Inhibitors of Autophagosome–Lysosome Fusion

Autophagosome–lysosome fusion is one of the critical steps during autophagy [77]. It occurs at the later stages of autophagy, and it can be inhibited by many agents such as Pulsatilla saponin D (PSD), liensinine, bafilomycin A1(Baf A1), and specific and potent autophagy inhibitor (spautin-1).

#### 6.3.1. Pulsatilla Saponin D (PSD)

PSD is a saponin derivative extracted from *Pulsatilla chinensis* (Bunge) Regel [274]. It inhibits autophagic flux through three mechanisms: initially, obstructing the fusion of autophagosomes with lysosomes; then elevating lysosomal pH; and finally, inhibiting the activation of lysosomal cathepsins. Notably, a study conducted in vitro explored the effects of PSD in combination with the alkaloid chemotherapeutic agent camptothecin (CPT) as well as its use as a single agent on human breast MCF-7 and MDA-MB-231 cancer cells. The results showed that PSD serves as an autophagy inhibitor, and whether combined with CPT or not, it led to an increase in p62 levels in both MCF-7 and MDA-MB-231 cells, confirming its effectiveness in autophagy inhibition [275,276].

#### 6.3.2. Liensinine

Another specific natural autophagy inhibitor is liensinine, which is an isoquinoline alkaloid extracted from the seed embryo of *Nelumbo nucifera* Gaertn. It has diverse biological activities, including preventing arrhythmias, reducing hypertension, preventing pulmonary fibrosis, and inducing relaxation in vascular smooth muscle. Studies indicate that liensinine inhibits autophagosome–lysosome fusion, causing the accumulation of autophagosomes and mitophagosomes. More specifically, it hinders autophagic degradation, blocks autophagosome–lysosome fusion, causes the accumulation of autophagy substrates, and delays the maturation of important lysosomal hydrolases. Furthermore, by inhibiting autophagy/mitophagy, liensinine enhances the susceptibility of breast cancer cells to the cell death-inducing effects of doxorubicin via DNM1L-dependent mitochondrial fission, suggesting its potential to synergize with chemotherapy in autophagy inhibition [125].

#### 6.3.3. Bafilomycin A1(Baf A1)

Baf A1 is a macrolide antibiotic isolated from *Streptomyces griseus*. It has a dual action by inhibiting vacuolar V-ATPase and interrupting the passage of proteins through the lysosomal membrane. It also blocks lysosomal acidification and autophagosome–lysosome fusion [277,278]. In addition, Baf A1 has demonstrated an ability to augment chemotherapy sensitivity in gastric, osteosarcoma, and colon cancer cells [279,280,281]. In the context of colon cancer, Baf A1 specifically targets the aberrant activity of mTOR, which contributes to cancer progression, chemotherapy resistance, and recurrence [279,280]. Moreover, activation of mTORC1/2 is essential to promoting cancer behaviors, such as tumor cell growth, survival, proliferation, and resistance to apoptosis [282,283]. When co-administered with WYE-354, potent inhibitors of mTORC1/2, Baf A1, and 3-MA enhance WYE-354’s anti-survival effect against HT-29 cells [284,285]. Additionally, the combination of Baf A1 and 3-MA increases the apoptotic activity of nedaplatin in cisplatin-resistant nasopharyngeal carcinoma cells by elevating LC3-II, cleaved caspase 3, and cleaved PARP levels in HNE1/DDP cells [286].

#### 6.3.4. Specific and Potent Autophagy Inhibitor Spautin-1

Another potent autophagy inhibitor, sapautin-1, is also worth being focused on. It is a derivative of quinazoline that enhances the downregulation of VPS34 complexes. By primarily targeting and inhibiting ubiquitin-specific peptidases such as USP10 and USP13, sapautin-1 affects the protein Beclin-1, leading to its degeneration during glucose-free conditions [93]. Consequently, autophagy is impeded because of the decrease of VPS34 [94]. Furthermore, the combined application of spautin-1 with imatinib mesylate (IM) could bring a synergistic therapeutic effect on chronic myeloid leukemia (CML) [94]. Acting as a selective agent against BCR-ABL [287,288], Imatinib (IM) helps in the prognostication of CML patients in the chronic phase. However, resistance to IM presents a challenge for patients in the progressive phase [287,289]. A study on synergistic effects revealed that spautin-1 increases the cytotoxic effect of IM in the K562 cell line (an immortalized myelogenous leukemia cell line) as well as in primary cells. It also suppressed IM-induced autophagy in a manner dependent on Beclin-1 and triggered the inactivation of PI3K/AKT while activating GSK3β, subsequently reducing the expression of the anti-apoptotic proteins Mcl-1 and Bcl-2. It is noteworthy that monotherapy of sapautin-1 does not significantly impact apoptosis, but it could increase IM-induced caspase-3 cleavage, indicating the enhancement of apoptosis [94].

Emodin [6-methyl-1,3,8-trihydroxy anthraquinone]. ROC-325 [1-((2-((2-((7-chloroquinolin-4-yl)amino)ethyl)(methyl)amino)ethyl)amino)-4-methyl-9H-thioxanthen-9-one]. Irinotecan [7-ethyl-10-hydroxycamptothecin]. 3-MA derivatives [4-(3-methyl-3H-purin-6-yl) thiomorpholine], [3-methyl-6-(3-methylpiperidin-1-yl)-3H-purine] and [6-(4-(3,4-dichlorophenyl) piperazin-1-yl)-3-methyl-3H-purine]. Spautin-1 [6-Fluoro-N-(4-fluorobenzyl) quinazoline-4 amine]. BAY 80-6946 [2-amino-n-(7-methoxy-8-(3-morpholinopropoxy)-2,3-dihydroimidazo[1,2-c]quinazolin-5-yl)pyrimidine-5-carboxamide]. Compound 31 [1-[4-[4-[4-(2,3-dihydro-1,4-benzodioxin-6-ylsulfanyl)-3-(trifluoromethyl)phenyl]pyridin-2-yl]piperazin-1-yl]ethanone]. CA-5f [3E, 5E0-3-(3,4-dimethoxybenzylidene)-5-[(1H-indol-3—yl)methylene]-1-methylpiperidin-4-one].

## 7. Gene Therapy Targeting Autophagy

In previous sections, the efficiency of antitumor compounds in regulating autophagy and suppressing cancer progression has been discussed. The next consideration is the availability of efficient genetic tools for suppressing the expression of ATGs at the transcriptional level. Gene silencers are specific DNA or RNA sequences that can hinder the expression of target genes, providing the potential to develop effective molecules with antitumor properties [290]. Studies have identified the upstream regulators of autophagy, and due to the utilization of genetic tools such as the CRISPR system and RNAi, molecular pathways can be targeted in autophagy regulation and affect the progression of cancers.

CRISPR-Cas9 can be used to create gene knockout models by introducing targeted double-strand breaks in the DNA of ATGs such as *ATG7*, *ATG5*, or *BECN1*, leading to the permanent loss of function of these genes and, thus, inhibiting autophagy [291,292,293,294,295]. On the other hand, RNAi utilizes short hairpin RNA (shRNA) or siRNA to silence gene expression post-transcriptionally. Specifically, by designing RNAi molecules to specifically target mRNA transcripts of key autophagy genes like *ULK1* or *VPS34*, the synthesis of these essential autophagy proteins can be effectively reduced [296]. Together, these techniques offer synergistic strategies for autophagy inhibition and potential therapeutic avenues: CRISPR-Cas9 causes permanent gene disruption, while RNAi allows for reversible gene silencing.

## 8. Clinical Trials Targeting Autophagy for Cancer Therapy

As previously mentioned, CQ and HCQ are the only FDA-approved drugs used as autophagy inhibitors. Notably, these drugs have demonstrated efficacy in the treatment of both COVID-19 and cancer, highlighting their potential therapeutic versatility. In COVID-19, they succeeded in blocking the viral infection through autophagy inhibition, though the clinical evidence is still controversial. Early studies suggested that these drugs could block SARS-CoV-2 infection in vitro. Additionally, an open-label non-randomized trial in France found that HCQ treatment alone significantly reduced COVID-19 patients’ viral load, with an enhanced effect when combined with azithromycin. However, this study had a small sample size. Larger observational studies, such as one involving over 1400 patients, found no benefit of HCQ in reducing the risk of death in hospitalized COVID-19 patients. Consequently, more recent clinical trial results have clearly demonstrated that CQ/HCQ alone or in combination with other agents did not show any benefit, leading the NIH to halt all clinical trials on these drugs and the FDA to strongly advise against their use for COVID-19 treatment [297]. Shifting to cancer treatment, their contribution in cancer therapy is still limited due to the complicated mechanisms of autophagy. Usually, autophagy inhibitors are utilized with cytotoxic agents because antitumor therapy creates intracellular stress and starves the cells. In this case, the addition of autophagy inhibitors could exacerbate the disruption of homeostasis in tumor cells, thus enhancing the efficacy of antitumor agents [298]. For instance, in a preclinical trial, the inhibition of autophagy by CQ or Baf A1 significantly strengthened the cytotoxic effect of pirarubicin on cervical cancer cells and inhibited tumor growth in the xenograft mouse model [299]. However, although autophagy inhibitors are involved in many preclinical studies, there are only limited data about them in clinical studies [300]. CQ and HCQ are both FDA-approved autophagy inhibitors evaluated in many cancer clinical trials [301]. Therefore, we conclude with information about clinical trials of some autophagy inhibitors, mainly focusing on CQ and HCQ in cancer therapy, aiming to gain a broad understanding of the current research status (Table 2).

### 8.1. CQ Monotherapy

A recent study investigated the potential efficacy of CQ in improving treatment outcomes for glioblastoma combined with chemoradiation [302,303,304,305]. The study is a Phase II randomized controlled clinical trial (ID: NCT02432417), and January 2025 is the final date for primary outcome measures. Two plans of treatment were included in this trial. Patients in the control group received a standard protocol of radiotherapy (30 daily fractions of 2 Gy or 33 fractions of 1.8 Gy) combined with temozolomide (75 mg/m^2^) daily, followed by six adjuvant cycles of temozolomide (150–200 mg/m^2^ daily by mouth). The other group used the same protocol except for the addition of CQ (400 mg/day), starting one week before radiotherapy and continuing until the last day. Nausea and fatigue were commonly reported side effects in this study, and most patients could fully recover after the treatment. In total, there were 11 serious adverse effects documented in eight patients, five of which were deemed unrelated to CQ. Two patients demonstrated a significant lengthening of the ECG QT-corrected interval during the study’s final week of treatment (CTCAE grade III). Although neither of these patients experienced any physical complaints due to cardiac conduction disturbances, more information about the drug safety in this experiment has yet to be updated. Despite the current trials and therapies, glioblastoma remains the most lethal type of brain cancer due to its resistance to surgery, radiation, and chemotherapy, with a median survival of only 14.6 months following diagnosis. Although there was a similarity in overall survival between patients taking CQ and those taking a placebo, the survival rate in the placebo group over time was about half that of the patients taking CQ. As a result, increasing the daily dose of CQ may be necessary to increase the efficacy of the combined therapy with temozolomide [302].

### 8.2. HCQ Monotherapy

The effectiveness of HCQ in treating patients who were diagnosed with metastatic pancreatic cancer was also evaluated. For instance, a phase II study comprised two arms, with the first arm receiving 400 mg (*n* = 10) and the second arm receiving 600 mg (*n* = 10) of HCQ orally twice daily. Patients continued taking the doses unless there were any adverse effects. The primary endpoint was two-month progression-free survival. Two (10%) of the 20 patients showed no disease progression at the two-month mark. The overall survival and median progression-free survival were 46.5 days and 69.0 days, respectively. Elevated alanine aminotransferase (*n* = 1) and lymphopenia (*n* = 1) are both grade 4 adverse effects of the treatment. The efficacy and tolerability in these two groups were comparable, which suggests that HCQ monotherapy achieves inconsistent autophagy inhibition and negligible therapeutic efficacy [306]. Thus, when similar clinical trials are to be conducted, further optimization is needed to maximize the effectiveness of HCQ. In another trial, HCQ was combined with dabrafenib (DAB) and trametinib (TRA) in a phase I/II study on mutant melanoma. Patients were randomly assigned to either receive DAB, TRA, and HCQ in combination (experimental group) or receive DAB and TRA with the option of adding HCQ when there was proven tumor progression (control group).

### 8.3. HCQ with Ulixertinib

Here are more examples of HCQ in the combined therapy for various kinds of tumors. In a current trial, HCQ is being combined with ulixertinib for gastrointestinal cancer [307]. Ulixertinib is a novel, potent ERK inhibitor that selectively blocks the MAPK signaling. It has demonstrated a potent effect in patients with tumors having alterations in that pathway. ClinicalTrials.gov has registered a non-randomized open-label phase II study with ID: NCT05221320. This study is an open-label, multi-center, phase II basket study. Ulixertinib is being administered with HCQ to patients with advanced gastrointestinal malignancy mutations in ERK or MAPK kinase, or who are harboring rat sarcoma virus, a member of the rapidly accelerated fibrosarcoma (non-V600 BRAF). The trial consists of five baskets based on the primary disease, including cholangiocarcinoma (intrahepatic, perihilar, or extrahepatic), pancreatic adenocarcinoma, colorectal adenocarcinoma, oesophagal adenoesophagal carcinoma or oesophagal squaoesophagal carcinoma, and gastroesophageal junction adenocarcinoma or gastric adenocarcinoma. The study starts on 26 May 2022, and is expected to be completed on 19 June 2024. It is being conducted in two stages and involves administering the same oral doses of 450 mg twice/day for ulixertinib and 600 mg twice/day for HCQ. Treatment cycles are repeated every 28 days. The objectives of this trial are to evaluate the response rate to the combination of ulixertinib and HCQ and any associated side effects [307].

### 8.4. HCQ with Sirolimus/Vorinostat

In the next phase of clinical examinations, sirolimus, vorinostat, and HCQ are investigated for their potential use in patients with advanced cancers. Sirolimus is an mTOR inhibitor approved for use in kidney transplant patients. Vorinostat is a histone deacetylase inhibitor approved by the FDA for cutaneous T-cell lymphoma. A phase I clinical trial continues to evaluate this combination’s safety, its antitumor effects, and the highest tolerable dose in patients with advanced cancers. This study, with ID: NCT01266057, is listed on ClinicalTrials.gov and involves 160 participants. There are two experimental groups in the study. Experimental group 1 comprises HCQ with sirolimus, starting with a dose of HCQ (200 mg/day) and sirolimus (2 mg/day). The cycle duration is 21 days. Experimental group 2 includes HCQ with vorinostat, starting with a dose of HCQ (200 mg/day) and vorinostat (200 mg/day). The cycle duration is also 21 days [308].

### 8.5. HCQ with Atezolizumab/Cobimetinib

An ongoing study aims to test the safety and efficacy of a combination of atezolizumab, cobimetinib, and HCQ for patients with advanced malignancies harboring a KRAS mutation, which contains errors that promote growth. This study is divided into two phases, with phase I utilizing the time-to-event continuous reassessment method to investigate the safety and the maximum tolerated dose of the combination treatment. The three dose levels involve different combinations of the three drugs. Dose 1 consists of HCQ (600 mg twice/day), cobimetinib (40 mg), and no atezolizumab. Dose 2 involves HCQ (600 mg twice/day), cobimetinib (40 mg), and atezolizumab (840 mg). Lastly, dose 3 consists of HCQ (600 mg twice/day), cobimetinib (60 mg), and atezolizumab (840 mg). All doses are administered orally except for atezolizumab, which is administered intravenously. Phase II will use the recommended phase I dose for three cohorts of KRAS mutation: (1) pancreatic adenocarcinoma, (2) colorectal adenocarcinoma, and (3) histology-agnostic adenocarcinoma. A total of 175 participants are enrolled in this active study, but potential participants are still being recruited, with an estimated end date in September 2024. While cobimetinib and atezolizumab are FDA-approved for other cancers, they have not been used to treat gastrointestinal cancer. The preliminary results have shown that the combination of these drugs effectively kills cancer cells and shrinks tumors in several KRAS-mutated cancers in animals. The second phase of the study will be amended after the preliminary safety and efficacy results from phase I [309].

### 8.6. HCQ with Paricalcitol

Also, HCQ and paricalcitol can be combined with gemcitabine and nab-paclitaxel in pancreatic cancer treatments. Paricalcitol, a form of vitamin D, blocks a signal in cancer cells responsible for the growth and spreading of the tumor cells. A phase II labeled and single-group assignment trial has been established for this drug combination to estimate its safety and the antitumor effect on metastatic pancreatic tumors. The protocol administration is as follows: HCQ (orally, twice/day), paricalcitol (IV, 3 times/week), and gemcitabine and nab-paclitaxel (IV, over 30 min) on days 1, 8, and 15. This trial is still being recruited and is expected to be completed by August 2024. Additionally, the primary outcome measure is the change in tumor size from baseline as measured by cross-sectional imaging at 8 weeks, while the secondary outcome measures are the incidence of adverse events, progression-free survival with a time frame of up to 3 years from the study start, and overall survival with a time frame up to 3 years from the study start [310].

### 8.7. HCQ with Abemaciclib

In another trial, abemaciclib (CDK4/6 inhibitor) and HCQ were tested in breast cancer patients. Furthermore, a phase II randomized controlled trial included 66 participants. The study assessed whether using HCQ and abemaciclib in combination to target the disseminated tumor cells (DTCs) in bone marrow could decrease or eliminate their number. Experimental arm A used abemaciclib only (150 mg twice/day), while experimental arm B used abemaciclib (100 mg or 150 mg twice/day) with HCQ (600 mg twice/day). Both drugs were taken orally. The primary outcomes for this study were the incidence of treatment-related adverse events during the first cycle of treatment (4 weeks) and the change in bone marrow DTCs after six cycles of therapy (approximately 6 months) compared to baseline. The study also evaluated the frequency of “clearance” of bone marrow DTCs by the arm after six cycles of treatment. A safety cohort of six patients at each dose of abemaciclib were assessed for protocol-defined “severe toxicity” during cycle 1 [311].

### 8.8. HCQ with Carfilzomib

Multiple myeloma (MM) is a destructive disease characterized by the secretion of large amounts of monoclonal immunoglobulin and the expansion of bone marrow plasma cells. Proteasome inhibitors have been used to target protein degradation in MM, but patients can become resistant to these drugs, and MM utilizes the autophagy mechanism for protein degradation. Preclinical studies have shown that combining carfilzomib and HCQ increases myeloma cell death and reverses MM cell resistance to carfilzomib. A phase I clinical trial has assessed the safety and efficacy of combination therapy with carfilzomib, dexamethasone, and HCQ in patients with relapsed/refractory MM. In this study, 19 patients were enrolled in a single-arm, dose-escalation trial at two centers, using a 3 + 3 design in five dose levels. All patients received a 14-day run-in with monotherapy of HCQ at their assigned dose level, followed by six 28-day HCQ/carfilzomib/dexamethasone cycles. The primary outcome measure was to estimate the maximum tolerated dose of HCQ when added to the standard-dose regimen of carfilzomib/dexamethasone, with secondary outcome measures including toxicity rates and efficacy assessments. This study was completed in December 2021, and no results have yet been posted on ClinicalTrials.gov as of September 2023. This study has important implications for developing new treatments for relapsed/refractory MM, and it could identify a new drug for patients with this disease [312].

### 8.9. HCQ with LY3214996

According to clinicaltrials.gov (NCT04386057), a clinical trial is assessing the efficacy and safety of the combination of LY3214996, an ERK inhibitor, and HCQ in patients with advanced pancreatic cancer. The trial is a phase II, open-label, randomized, two-arm study with a safety lead-in. The study has enrolled 52 participants randomized 1:1 to receive either the combination of LY3214996 and HCQ or monotherapy with LY3214996. The primary outcome measure is the disease control rate, while the secondary outcome measures include the objective response rate, progression-free survival, overall survival, and dose-limiting toxicity. Participants are given the drugs on an outpatient basis, and treatment is administered continuously throughout each 28-day cycle. This study’s expected primary completion date is 18 November 2023, while the estimated study completion date is 19 December 2023. The trial was initiated on 27 May 2020, and the US FDA has not approved LY3214996 as a treatment for any disease [313].

Currently, only CQ and HCQ have been examined as autophagy inhibitors in clinical trials for cancer treatment, and most studies are still in phase I or II. Additionally, the clinical benefits of single and combinatorial treatments have not been conclusively demonstrated yet, and many clinical trials are still ongoing or planned. Therefore, more studies should be conducted to investigate the potential positive effect of autophagy inhibitors, providing cancer patients with better treatment options in the future.

## 9. Conclusions and Perspectives

Autophagy is a fundamental cellular process involved in the degradation and recycling of the components of the cell. It plays a complex, microenvironment-dependent role in cancer. Sometimes, this process protects established tumors in the early stages, presenting a convincing rationale for targeting autophagy in cancer therapy. For this reason, various autophagy inhibitors targeting different steps of the autophagy pathway have been developed and evaluated in preclinical studies and early clinical trials. These inhibitors have shown potential efficacy and initial safety, especially when combined with other drugs. However, numerous questions remain regarding optimal dosing, timing, and integration into multi-modal cancer treatment programs, which need further investigations.

Additionally, personalized medicine is an emerging approach to maximize autophagy inhibition therapy by tailoring strategies to specific cancer biochemical profiles and genotypes. Furthermore, the study of autophagy and drug-targeting opportunities is driven by developing new technologies and methodologies like high-throughput drug screening, monitoring autophagy signaling, advanced microscopy, genetic analysis, and computational approaches. While substantial progress has clarified the role of autophagy in cancer and paved the way for therapeutic targeting, significant research gaps remain to be addressed. Continued efforts to improve the integration of autophagy manipulation into oncology, guided by both emerging science and ethical principles, will be key steps for realizing the full potential of this promising new approach and improving cancer treatment and outcomes.

## Figures and Tables

**Table 1:** Subset of autophagy inhibitors for anticancer preclinical research.

Targets	Inhibitors	The Combination	The Molecular Mechanisms of Anticancer Action	References
Class III PI3K	KU55933	Gefitinib	Blocking the activity of ABCG2 in colorectal cancer	[82,83,84]
	Gö6976	Retinoic acid	Inhibiting DNA damage-induced G2 arrest and reverting metastatic phenotype in aggressive melanoma by reversing the E- to N-cadherin switch	[85,86,87,88]
	AZ7328	-	Inhibiting proliferation and AKT substrate phosphorylation in bladder cancer cells	[89]
	AZD5363	CQ	Inducing apoptosis and delaying tumor progression in prostate cancer cells	[90]
	RAB5A	-	Blocking autophagy by activating mTOR pathway in gastric cancer cells	[91]
	Lipoic Acid	-	Blocking autophagy by activating mTOR pathway in lung cancer cells	[92]
	Oxymatrine	Doxorubicin	Activating PI3K/AKT/ mTOR pathway	[79]
VPS34	Spautin-1	Imatinib (IM)	Inhibiting IM-induced autophagy in a Beclin-1-dependent manner in the K562 cells	[93,94,95,96]
	PIK-III	-	Blocking de novo lipidation of the microtubule-linked protein 1-light chain 3 (LC3)	[97,98,99]
	Compound 31	-	blocking VPS34 and mTORC1 signaling	[100,101]
	VPS34-IN1	-	blocking VPS34 and mTORC1 signaling	[102,103]
	SAR405	Everolimus	Reducing cell proliferation in renal cancer cells	[104,105]
Autophagy flux	Lucanthone	Temozolomide	Inducing lysosomal membrane permeabilization Enhancing TMZ efficacy in glioma stem cells	[106,107]
	Toosendanin (TSN)	Irinotecan	Deacidifying lysosome in triple-negative breast cancer cells (TNBC)	[80]
	4-Acetylantroquinon B	Cisplatin	Inhibiting PI3K/AKT/mTOR/p70S6K signaling pathway	[108]
	ARN5187	-	Blocking the late stage of autophagy	[109,110]
	Ganoderma Lucidum polysaccharide	CQ	Inducing autophagosome accumulation and apoptosis	[111,112,113]
	Oxautin-1		Inhibiting autophagosome biogenesis and maturation	[114]
	Tambjamine	-	Inducing lysosomal deacidification	[115]
	Dauricine and daurisoline	Camptothecin (CPT)	Inhibiting the lysosome V-type ATPase activity Inhibiting lysosomal degradation of autophagic vacuoles in HeLa cancer cells	[116]
	Berbamine	Icotinib	Inducing autophagosome accumulation by blocking autophagosome–lysosome fusion in lung cancer cells	[81,117]
	Monensin	Rapamycin	Disrupting lysosome acidification Enhancing cell cycle arrest and apoptosis induced by mTOR or EGFR inhibitors in lung cancer cells	[118,119,120,121]
	Madangamines	-	Inhibiting lysosomal function by increasing lysosomal pH	[122]
	Elaiophylin	Cisplatin	Inhibiting autophagic flux at the late stage of autophagy in ovarian cancer cells	[123]
	Pulsatilla saponin D (PSD)	CPT	Blocking the autophagosome–lysosome fusion process, elevating lysosomal pH and inhibiting lysosomal cathepsins activation in human breast cancer cells	[72]
	Schizocapsa plantaginea Hance 1	-	Inhibiting autophagosome–lysosome fusion	[124]
	Liensinine	Doxorubicin	Inhibiting autophagy/mitophagy and sensitizing breast cancer cells to doxorubicin through DNM1L-dependent mitochondrial fission	[125]
	Z- Ligustilide	Tamoxifen	Inhibiting autophagy and accumulating DNA damage in breast cancer cells	[126]
	Ginsenoside	Cisplatin	Activating AMPK and attenuating mTOR phosphorylation Inducing apoptosis and enhancing cell cycle alterations in bladder cancer cells	[67,72]
	[6]-Gingerol	Cisplatin	Reducing the expression of VEGF, FLT1, KDR, and Bcl-2 genes in ovarian cancer cells Inhibiting AMPK and AKT/mTOR signaling pathways	[127,128]
	Jolkinolide B	-	Inhibiting mTOR-induced AKT feedback activation	[129]
	Misaponin B	-	Inducing G2/M arrest and cytokinesis failure	[130]
	Clomipramine	-	Blocking autophagolysosomal fluxes	[131,132,133]
	Chloroquine (CQ)	Tetrandrine	Blocking autophagosome fusion and degradation in lung and liver cancer cells	[67,72]
	Hydroxychloroquine (HCQ)	Resveratrol	Blocking autophagosome fusion and degradation in osteosarcoma cells	[134]
	Quinacrine	Cediranib	Accumulating autophagic vacuole and leading to apoptosis in intracranial mouse glioma	[67,72,73,135]
	Lys05	-	Deacidifying the lysosome and blocking the late stage of autophagy	[136,137]
	Compound 30	-	Blocking autophagosome formation	[110,138]
	Verteporfin	-	Blocking autophagosome formation	[139,140,141]
	Clarithromycin	5-fluorouracil (5-FU)	Modulating the autophagic flux leads to apoptosis in colorectal cancer cells	[142,143,144]
	DQ661	Gemcitabine	Targeting protein-palmitoyl thioesterase 1 and affecting lysosomal function in pancreatic cancer cells	[145,146]
	VATG-027/VATG-032	-	Deacidifying the lysosome and disrupting the autophagosome	[147]
	Mefloquine	-	Disrupting autophagic flux by inducing mitochondrial autophagy	[148]
	WX8 family	-	Disrupting lysosome fission by tubulation and increasing the trafficking of molecules in lysosomes without elevating lysosomal acidity	[149]
	Vacuolin-1	-	Inducing the accumulation of autophagosomes by activating RAB5A	[150,151]
	Desmethylclomipramine	Doxorubicin	Blocking autophagic flux and sensitizing cells to cytotoxic agents	[133,152]
	ROC-325	Azacitidine (AZA)	Increasing autophagosome accumulation in acute myeloid leukemia	[153,154]
	Trifluoperazine	-	Inducing G0/G1 arrest and apoptosis	[155,156]
	Squaramides	-	Arresting the cell cycle at phase G1 and caspase-dependent apoptosis	[157,158]
	FV-429	-	Blocking autophagic flux and induced autophagosome accumulation	[159,160,161]
	GNS561/Ezurpimtrostat	-	Inhibiting the late stage of autophagy by inducing lysosomal dysregulation	[162,163]
	Pantoprazole	Docetaxel	Inhibiting acidification of endosomes or autophagosome–lysosome fusion	[164,165]
	LAI-1	Cisplatin	Inducting lysosomal dysfunction and blocking autophagy–lysosome formation in lung cancer cells	[166]
	Tambjamines (anion-selective ionophores)	-	Blocking autophagy by inducting of lysosomal dysfunction	[167]
	IITZ-01 and IITZ-02	-	Blocking autophagy by disrupting lysosomal enzymes and pH in TNBC	[168]
	CUR5g	Cisplatin	Blocking the recruitment of STX17 to autophagosomes via a UVRAG-dependent mechanism in NSCLC cells	[166,169]
V-ATPase	Bafilomycin A1 (Baf A1)	CQ	Decreasing mitochondrial quality and bioenergetic function in primary neurons	[170,171,172,173,174]
	Concanamycin A	Vorinostat	Inducing apoptosis in oral squamous cell carcinoma cells	[175,176]
ATGs	NSC185058	-	Inhibiting ATG4B enzymatic activity and blocking autophagic flux	[177,178]
	Tioconazole	Nicardipine	Inhibiting autophagy and promoting cell death in glioma stem cells	[37,179,180]
	LV-320	-	Inhibiting ATG4B enzymatic activity and blocking autophagic flux	[181]
	S130	Caloric restriction	Inhibiting ATG4B and inducing cell death in colorectal cancer cells	[182]
ULK	ULK-101	KRAS-targeted drug (AMG-510)	Inhibiting autophagy induction and autophagic flux in NSCLC cells	[33,183,184,185]
	ULK-100	-	Inhibiting autophagy induction and autophagic flux	[33,183,184]
	MRT67307	Amino acid withdrawal	Blocking mTOR-dependent autophagy	[186]
	SBI-0206965	Cisplatin/AZD8055	Blocking cisplatin-induced autophagy and promoting cell death Inhibiting AMPK and increasing apoptosis in lung cancer cells	[33,187,188,189]
	Compound 6	-	Inhibiting autophagy by blocking ULK 1/2	[190]
	MRT68921	WZ4003	Inhibiting autophagy by blocking ULK 1 and NUAK1	[191]
	DCC-3116	Encorafenib and Cetuximab	Blocking autophagosome formation and ULK1/2 protein kinase in colorectal cancer cells	[192,193]
	XST-14	Sorafenib	Suppressing the invasion and proliferation of hepatocellular carcinoma cells by blocking ULK1 activity	[194]
	MiR-93	CQ or NSC185085	Downregulating BECN1, ATG5, ATG4B, and SQSTM1 proteins in glioblastoma cells	[195]
	SR-17398	-	Inhibiting autophagy by blocking ULK1	[196]
Acid proteases	Pepstatin A	E64d	Inducing autophagolysosome accumulation	[197,198,199,200,201,202,203]
	Leupeptin	Cycloheximide	Inhibiting the autophagic vacuole formation and the sequestrations of cytoplasmic and lysosomal enzymes in pancreatic acinar cells	[203,204,205]
Tyrosine kinase	IM	CQ/Clarithromycin/BafA1	Inhibiting Hedgehog signaling pathway and overcoming drug resistance of BCR-ABL-positive chronic myeloid leukemia (CML) cells Increasing ROS production	[206,207,208,209]
	Sorafenib	CQ/3-MA	Blocking autophagy, reducing intracellular energy synthesis, and causing lipid accumulation in hepatocellular carcinoma cells Impairing autophagy via ATG4B inhibition in glioblastoma cells	[210,211,212]
	Sunitinib	CQ/Bromodomain	Inducing apoptosis in renal cancer cells Inhibiting autophagy through GDF15 suppression in melanoma cells	[213,214,215]
	Linifanib	CQ, HCQ or 3-MA	Inhibiting autophagy and sensitizing hepatocellular carcinoma cells to linifanib	[216]
	Gefitini	HCQ/BafA1/3-MA, Clarithromycin/EGCG/CQ	Inducing mitochondrial apoptosis in TNBC cells Inhibiting autophagy through targeting ERK phosphorylation in NSCLC cells	[217,218,219,220,221]
	Erlotinib	Shikonin/CQ	Overcoming the innate resistance of wild-type EGFR in NSCLC cells to erlotinib Inhibiting autophagy in lung cancer cells through modulating endoplasmic reticulum stress	[222,223]
	Cediranib	Quinacrine	Enhancing anti-vascular and antitumor efficacy of cediranib in intracranial mouse glioma cells	[224]
	Carfilzomib	Emodin/CQ/HCQ	Increasing cellular ROS production and inducing apoptosis in multiple myeloma cells	[225,226,227]
Proteasome	Bortezomib	Solamargine/BafA1/CQ, 3-MA/ATG7 siRNA	Inducing autophagy-mediated apoptosis and enhancing bortezomib activity in multiple myeloma cells Inhibiting autophagosome–lysosome fusion by blocking acidification Enhancing apoptosis in human glioblastoma cells and enhancing bortezomib activity in multiple myeloma cells	[228,229,230,231,232]
	Ixazomib	ABT-737 Doxorubicin	Inhibiting autophagy and MCL-1 expression sensitizes colorectal cancer cells to ixazomib Inhibiting proteasome and autophagy sensitizes breast cancer cells to doxorubicin	[233,234]

**Table 2:** Overview of clinical trials on autophagy inhibitors for cancer therapy.

Trail ID	Condition	Status of Study	Treatment	Phase	Outcome Measures	Methods		
						Doses	Duration	Single/Combined
NCT03754179	Melanoma	Recruiting	Dabrafenib (DAB) Trametinib (TRA) Hydroxychloroquine (HCQ)	1, 2	Safety, efficacy	DAB (150 mg/day) TRA (2 mg/day) HCQ (200 mg twice/day)	Started in January 2018 and completed in July 2022	Combined
NCT02432417	Glioblastoma, astrocytoma (Grade IV)	Withdrawn	CQ Temozolomide Chemoradiation	2	Overall survival, side effects, tumor hypoxia	CQ (400 mg/day) Temozolomide (75 mg/m^2^) Radiation (30 fractions of 2 Gy)	Started in November 2023, and will complete in November 2023	With radiotherapy
NCT05221320	Advanced gastrointestinal malignancies (RAS mutation)	Recruiting	Ulixertinib HCQ	2	Overall response rate, side effects	Ulixertinib (450 mg BID twice/day) HCQ (600 mg BID/day)	Started in May 2022, and will complete March in 2025	Combined
NCT01266057	Advanced cancer types	Completed	HCQ Sirolimus Vorinostat	1	Estimated maximum tolerated dose, safety, efficacy	HCQ (200 mg/day) Sirolimus (2 mg/day) Vorinostat (200 mg/day)	Started in April 2011 and completed in February 2021	Combined
NCT04214418	Gastrointestinal, pancreatic, and agnostic cancer (specifically, KRAS-mutated advanced malignancies)	Active, not recruiting	HCQ Cobimetinib (MEK inhibitor) Atezolizumab (Immune Checkpoint Blockade)	1, 2	Estimated maximum tolerated dose, safety, tolerability	HCQ (600 mg twice/day) Cobimetinib (40–60 mg) Atezolizumab (840 mg/day 1 and 15)	Started in February 2020, and will complete in September 2024	Combined
NCT04524702	Advanced pancreatic cancer	Active, not recruiting	Paricalcitol HCQ Gemcitabine and Nab-paclitaxel	2	Antitumor effect, safety	Paricalcitol (IV 3 times/week) HCQ (twice a day/month) Gemcitabine and Nab-paclitaxel IV (Over 30 min/days 1, 8, and 15)	Started in September 2020, and will complete in August 2024	Combined
NCT04386057	Pancreatic cancer (metastatic)	Active, not recruiting	HCQ LY3214996	2	Safety, antitumor activity	HCQ (Twice/day by mouth) LY3214996—not stated	Started in May 2020, and will complete in February 2024	Combined
NCT04523857	Breast cancer	Recruiting	Abemaciclib HCQ	2	Safety, efficacy	Abemaciclib (100–150 mg twice/day) HCQ (600 mg twice/day)	Started in November 2021, and will complete in December 2028	Combined
NCT00765765	Breast cancer	Terminated	HCQ Ixabepilone	1, 2	Tumor response rate, survival rate, biomarkers of autophagy inhibition	HCQ (200 mg/day/month to 200 mg twice/day/month) Ixabepilone dose range is 32–40 mg/m^2^	Started in February 2009 and completed in December 2011	Combined
NCT03774472	ER-positive HER2-negative breast cancer	Active, not recruiting	HCQ Palbociclib Letrozole	1, 2	Dose response, cancer cell proliferation, cell cycle	HCQ (400 mg versus recommended phase 2 dose) Palbociclib and Letrozole—not stated	Started in August 2018, and will complete in December 2025	Combined
NCT04163107	Advanced multiple myeloma	Completed	HCQ Carfilzomib Dexamethasone	1	Maximum tolerated dose	To be defined	Started in July 2020 and completed in December 2021	Combined
NCT00568880	Myeloma	Completed	HCQ Bortezomib	1	Safety, efficacy	HCQ (200–600 mg/day) Bortezomib IV (1.0–1.3 mg/m^2^)	Started in September 2010 and completed in June 2011	Combined
NCT04892017	Pancreatic ductal adenocarcinoma, melanoma, non-small cell lung cancer, colorectal cancer, and solid metastatic tumor	Recruiting	DCC-3116 TRA Binimetinib Sotorasib	1, 2	Tumor response rate	DCC-3116 (orally twice/day) TRA, binimetinib, and sotorasib—not stated	Started in June 2021, and will complete in October 2024	Monotherapy and combination
NCT04911816	Pancreatic adenocarcinoma	Recruiting	mFOLFIRINOX HCQ	1, 2	Maximum tolerated dose of FHQ, tumor response rate	mFOLFIRINOX—not stated, HCQ orally (400–1200 mg)	Started in July 2021, and will complete in June 2028	Combined
NCT04566133	Bile tract carcinoma (KRAS mutation refractory)	Completed	HCQ TRA	2	Progression free survival, response rate, safety	HCQ orally (600 mg twice/day) TRA orally (2 mg/day)	Started in February 2015 and completed in December 2022	Combined
NCT02337309	Neuroblastoma	Terminated	SF1126	1	Safe dose in the pediatric population	SF1126 IV (3 + 3 dose escalation)	Started in July 2015 and completed in May 2018	Monotherapy
NCT03037437	Hepatocellular cancer	Recruiting	Sorafenib HCQ	2	Time to tumor progression	Sorafenib (400 mg/day) HCQ (400 mg/day)	Started in February 2017, and will complete in March 2025	Combined
NCT05576896	Stage IV colorectal (BRAF V600E)	Recruiting	HCQ Encorafenib Cetuximab Panitumumab	2	Tumor response, survival, safety	HCQ—not stated Encorafenib (300 mg/day) Cetuximab (250 mg/m^2^–400 mg/m^2^) Panitumumab—not stated	Started in October 2022, and will complete in July 2025	Combined
NCT05036226	Advanced solid tumors or relapse prostate cancer	Recruiting	HCQ Metformin Sirolimus Dasatinib and nelfinavir	1, 2	Maximum tolerated dose, quality of life, disease control rate	HCQ (600mg twice/day) Metformin (500 mg/day for 7 days, then increase to 1000 mg/daily) Sirolimus (0.5 mg/day) Dasatinib and nelfinavir—not stated	Started in March 2022, and will complete in October 2025	Combined
NCT05070104	Metastatic colorectal cancer	Withdrawn (no funding)	CPI-613 (Devimistat) Modified FFX Bevacizumab	1	Safety and tolerability, objective response rate, overall survival	CPI-613 (250–1000 mg/m^2^) Irinotecan (50 mg/m^2^), Leucovorin (400 mg/m^2^), Oxaliplatin (85 mg/m^2^), 5FU (2400 mg/m^2^) Bevacizumab (5 mg/kg)	Started in March 2023, and will complete in November 2024	Combined
NCT05708326	Chronic lymphocytic leukemia, small lymphocytic lymphoma	Com-pleted	Intermittent Fasting	A Case Crossover Study	Changes in lymphocytes, metabolites, autophagy, gene expression, inflammation, gut microbiome	5:2 Method (intermittent fasting regimen) 16/8 Method (intermittent fast regimen)	Started in June 2023, and completed in March 2024	-
NCT04527549	Melanoma (Stage IIIC or IV BRAF V600 E/K)	Active, not recruiting	DAB TRA with or without HCQ	2	Progression-free survival, overall survival, adverse events, treatment duration	To be defined	Started in June 2021, and will complete in November 2025	Combined
NCT05763992	Triple-negative breast cancer	Recruiting	Chemoimmunotherapy and fasting	2	Pathologic response, survival, safety, compliance, adverse events	Control diet or fasting-like approach Anthracycline–taxane–carboplatin chemotherapy plus pembrolizumab	Started in May 2023, and will complete in May 2026	-
NCT00813423	Advanced solid tumors (not responded to chemotherapy)	Completed	Sunitinib Malate HCQ	1	Dose response, survival, efficacy, biomarkers, safety	To be defined	Started in February 2010 and completed in July 2023	Combined and monotherapy
NCT04841148	Breast cancer ER positive (disseminated tumor cells)	Recruiting	Avelumab or HCQ with or without palbociclib	2	Safety, efficacy, recurrence	Avelumab (10 mg/kg) HCQ (600 mg twice/day) Palociclib (125 mg/day)	Started in June 2021, and will complete in May 2028	Combined
NCT02512926	Children with solid tumors (relapsed/refractory) or leukemia	Completed	Carfilzomib Cyclophosphamide Etoposide	1	Dose response, toxicity, biomarkers, genomic predictors	To be defined	Started in February 2016, and completed in January 2024	Combined
NCT05448677	Hepatocellular carcinoma (unresectable)	Recruiting	Ezurpimtrostat Atezolizumab Bevacizumab	2	Progression-free survival, objective response rate, tumor response	Ezurpimtrostat—not stated Atezolizumab (1200 mg/day) Bevacizumab (15 mg/kg)	Started in December 2022, and will complete in December 2025	Combined
NCT03598595	Osteosarcoma (recurrent or refractory)	Active, not recruiting	Gemcitabine Docetaxel HCQ	1, 2	Maximum tolerated dose, disease control rate, event-free survival, overall response	To be defined	Started in January 2019, and will complete in September 2024	Combined
NCT03529448	Glioblastoma	Recruiting	TN-TC11G (THC + CBD) Temozolomide Radiation	1, 2	Dose, efficacy, safety, survival, biomarkers	TN-TC11G—not stated. Temozolomide (75 mg/m^2^, 150 mg/m^2^, 200 mg/m^2^) Radiation (1.8–2.0 Gy/day) (total dose 58–60 Gy)	Started in July 2023, and will complete in December 2025	Combined
NCT04201457	Glioma of the brain	Recruiting	DAB TRA HCQ	1, 2	Dose, efficacy, PK, safety, biomarkers, progression	To be defined	Started in January 2020, and will complete in June 2029	Combined
NCT02339168	Hormone-resistant prostate cancer	Active, not recruiting	Enzalutamide Metformin Hydrochloride	1	Toxicity, efficacy, PSA response, survival, radiographic progression	To be defined	Started in June 2016, and will complete in December 2024	Combined
NCT01480154	Advanced solid tumors (melanoma, prostate or kidney cancers)	Active, not recruiting	MK2206 (inhibitor) and HCQ	1	Toxicity, dose, autophagy biomarkers	To be defined	Started in November 2011, and its primary completion date was February 2020	Combined
NCT04132505	Metastatic pancreatic cancer (KRAS mutation)	Recruiting	Binimetinib HCQ	1	Dose, efficacy, safety, survival, biomarkers, body composition changes	To be defined	Started in October 2019, and will complete in December 2023	Combined
NCT04873895	Metastatic colorectal cancer (liver dominant)	Recruiting	TACE (transarterial chemoembolization) Axitinib HCQ	1	Safety, liver response, progression-free overall survival	TACE (4–8-week intervals) Axitinib (5 mg twice/day) HCQ (600 mg twice/day)	Started in January 2022, and will complete in December 2024	Combined
NCT04190433	Lymphoma, sarcoma, breast cancer	Withdrawn	Anthracycline Lisinopril Pravastatin Spironolactone	2	Cardiac function, recovery rates, time to recovery	To be defined	Started in September 2020 and completed in April 2023	Combined
NCT02316340	Colorectal cancer	Completed	Vorinostat HCQ Regorafenib	2	Progression-free survival	Vorinostat (400 mg by mouth/day) HCQ (600 mg by mouth/day) Regorafenib (160 mg by mouth/day)	Started in February 2015 and completed in April 2018	Combined
NCT01206530	Rectal cancer, colon cancer metastasis	Completed	HCQ Oxaliplatin Leucovorin 5-FU Bevacizumab	1, 2	progression-free survival, overall survival, toxicity incidence, autophagy markers	HCQ (600 or 800 mg) Oxaliplatin (85 mg/m^2^) Leucovorin (400 mg/m^2^) 5-FU (400–2400 mg/m^2^) Bevacizumab—not stated	Started in September 2010 and completed in September 2017	Combined
NCT01978184	Pancreatic cancer	Completed	Gemcitabine Abraxane HCQ	2	Histopathologic response, survival, toxicity	Gemcitabine (1000 mg/m^2^) Abraxane (125 mg/m^2^) HCQ (1200mg)	Started in November 2013 and completed in February 2018	Combined
NCT01510119	Metastatic clear cell renal cell carcinoma	Completed	RAD001 HCQ	1, 2	Disease control	RAD001 (10 mg/day by mouth) HCQ (400 mg twice/day by mouth)	Started in September 2011 and completed in January 2017	Combined
NCT02257424	Advanced BRAF mutant melanoma	Completed	HCQ TRA DAB	1, 2	Maximum tolerated dose, progression-free survival rate	HCQ—not stated TRA (2 mg/day) DAB (150 mg twice/day by mouth)	Started in October 2014 and completed in October 2021	Combined
NCT01023477	Breast cancer	Completed	CQ	1, 2	Tumor size, cancer cell proliferation index, treatment-related adverse events	CQ (250 mg/week in phase 1) CQ (500 mg/week in phase 2)	Started in December 2009 and completed in October 2016	Monotherapy
NCT01777477	Pancreatic cancer	Completed	CQ Gemcitabine	1	Maximum tolerated dose	CQ (100 mg, 200 mg or 300 mg) Gemcitabine (1000 mg/m^2^)	Started in July 2012 and completed in May 2015	Combined
NCT01469455	Melanoma	Completed	DT01CQ	1	Tolerability, safety, pharmacokinetics	DT01 (16–64 mg/3 times a week) CQ—not stated	Started in October 2011 and completed in July 2015	Combined
NCT02378532	Glioblastoma	Completed	CQ Temozolomide Radiotherapy	1	Toxicity, pharmacokinetics, maximum tolerated dose, autophagic markers, EGFRvIII status	CQ (200–600 mg) Temozolomide (75 mg/m^2^)	Started in August 2016 and completed in July 2019	Combined
NCT04397679	Glioblastoma	Recruiting	CQ Temozolomide Radiotherapy	2	Overall adverse events, incidence of dermatitis toxicity	To be defined	Started in August 2021, and will complete in April 2025	Combined
NCT01438177	Multiple myeloma	Terminated	CQ Velcade Cyclophosphamide	2	Response rate, adverse events, response relative to autophagy	CQ (500 mg/day by mouth) Velcade (1.3 mg/m^2^) Cyclophosphamide (50 mg twice/day by mouth)	Started in October 2011 and completed in February 2014	Combined
NCT01006369	Colorectal cancer	Completed	HCQ	2	Progression-free survival, overall response rate, safety, disease control rate, response duration, autophagy biomarkers	HCQ (200 mg/day)	Started in May 2009 and completed in April 2016	Combined with capecitabine, oxaliplatin, and bevacizumab
NCT01978184	Pancreatic cancer	Completed	HCQ Gemcitabine Abraxane	2	Histopathology, survival, treatment response	HCQ (1200 mg twice/day) Gemcitabine (1000 mg/m^2^) Abraxane (125 mg/m^2^)	Started in November 2013 and completed in February 2018	Combined
NCT00224978	Glioblastoma	Completed	CQ	3	Survival after surgery, survival at two years	To be defined	Started in January 2005, and its completion date was in August 2005	Monotherapy
NCT01446016	Breast cancer	Completed	CQ Paclitaxel Docetaxel Abraxane Ixabepilone	2	Overall response rate, progression-free survival, overall survival	CQ (250 mg/day) Paclitaxel-175 mg/m^2^ Docetaxel (75 mg/m^2^) Abraxane (260 mg/m^2^) Ixabepilone (40 mg/m^2^)	Started in September 2011, and its completion date was in March 2019	Combined
NCT02496741	IDH1/2-mutated solid tumors	Completed	CQ Metformin	1, 2	Maximum tolerated dose, D2HG concentration in serum/urine/bile/ tumor, tumor response, dose response	CQ (once/day) Metformin (twice/day)	Started in November 2015 and completed in November 2019	Combined
NCT02232243	Solid tumor	Completed	HCQ	1	Number of patients with elevated Par-4 levels, optimal HCQ dose based on Par-4 toxicity and response	HCQ (200 mg twice/day)	Started in July 2015 and completed in December 2018	Monotherapy
NCT01273805	Pancreatic cancer	Completed	HCQ	2	Progression-free survival rate, tumor response rate, overall survival, toxicity	HCQ (600 mg twice/day by mouth)	Started in January 2011 and completed in February 2014	Monotherapy
NCT01649947	Lung cancer	Completed	HCQ Paclitaxel Bevacizumab Carboplatin	2	Tumor response rate, progression-free survival, overall survival, adverse events	HCQ (400 mg/day) Paclitaxel (200 mg/m^2^) Bevacizumab (15 mg/kg) Carboplatin IV (over 15–30 min)	Started in December 2011 and completed in June 2015	Combined
NCT01506973	Pancreatic cancer	Completed	HCQ Gemcitabine Abraxane	1, 2	Overall and one year of survival	HCQ (1200 mg/day) Gemcitabine (1000 mg/m^2^) Abraxane (125 mg/m^2^)	Started in December 2011 and completed in March 2022	Combined
NCT00486603	Central nervous system tumors	Completed	HCQ Temozolomide Radiation	1, 2	Maximum tolerated dose, pharmacokinetics, overall survival, autophagy inhibition, toxicity, correlations with genetic markers	HCQ (200 mg/day) Temozolomide (75 mg/m^2^)	Started in October 2007 and completed in January 2014	Combined
NCT02071537	Malignant neoplasm	Completed	CQ Gemcitabine Carboplatin	1	Maximum tolerated dose, overall survival, time to disease progression	CQ (50–200 mg/day) Gemcitabine (1250 mg/m^2^) Carboplatin—not stated	Started in December 2014 May and completed in December 2018	Combined
NCT03513211	Prostate cancer	Completed	HCQ Suba-itraconazole	1, 2	Dose, efficacy, safety, disease progression	HCQ (Escalating doses in Rolling 6 Phase I) Suba-itraconazole (150 mg PO BD)	Started in August 2018, and will complete in October 2023	Combined
NCT03344172	Pancreatic cancer	Terminated	Gemcitabine Nab-Paclitaxel HCQ Avelumab	2	Safety, histopathologic response, changes in autophagy biomarkers, coagulation index	Gemcitabine (1000 mg/m^2^) Nab-paclitaxel (125 mg/m^2^) HCQ (600 mg/BID/day) Avelumab (10 mg/kg)	Started in December 2017 and completed in April 2019	Combined
NCT00726596	Prostate cancer	Completed	HCQ	2	PSA response, safety, autophagy biomarkers	HCQ (400–600/day)	Started in August 2008 and completed in January 2018	Monotherapy
NCT05680662	Metastatic breast cancer and TNBC	Not yet recruiting	Quercetin EGCG Metformin Zinc	1	Invasive disease-free survival, adverse events	Quercetin (500 mg/ day) EGCG (300 mg/day) Metformin (850 mg/day) Zinc (50 mg/day)	Started in January 2023, and completed in January 2024	Combined
NCT01128296	Pancreatic cancer	Completed	HCQ Gemcitabine	1, 2	Efficacy, safety	HCQ (200–1200 mg/day) Gemcitabine (10 mg/m^2^/min)	Started in October 2010 and completed in July 2014	Combined
NCT04011410	Prostate cancer	Active, not recruiting	HCQ	2	Cancer progression, ADT-free survival, progression-free survival	HCQ (200 mg twice/day by mouth)	Started in December 2019, and will complete in November 2026	Monotherapy
NCT01550367	Renal cell cancer	Completed	HCQ Interleukin-2	1, 2	Efficacy, safety, immune response	HCQ (600 mg/day) Interleukin-2 (600,000 IU/kg)	Started in March 2012 and completed in February 2019	Combined
NCT04735068	Non-small cell lung cancer, KRAS mutation-related tumors	Active, not recruiting	HCQ Binimetinib	2	Safety, efficacy	HCQ (400 mg twice/day) Binimetinib (45 mg twice/day)	Started in April 2021, and will complete in December 2023	Combined
NCT05083780	Pancreatic cancer	Active, not recruiting	HCQ Chlorphensin carbamate mFOLFIRINOX	1	Safety, efficacy	HCQ (200 mg twice/day by mouth) Chlorphenesin carbamate (250 mg twice/day by mouth) mFOLFIRINOX—not stated	Started in November 2021, and will complete in December 2024	Combined
NCT05518110	Pancreatic cancer	Recruiting	HCQ TRA	2	Safety, efficacy	HCQ (600 mg twice/day by mouth) TRA (2 mg/day by mouth)	Started in May 2023, and will complete April 2025	Combined
NCT03979651	Melanoma	Completed	HCQ TRA	1, 2	Safety, efficacy, survival, side effects	HCQ (400 mg once/day by mouth) TRA (2 mg once/day by mouth)	Started in October 2019, and its completion date was in March 2022	Combined
NCT05953350	HR+/HER2− breast cancer	Recruiting	HCQ Palbociclib	1, 2	Safety, efficacy, dose response, survival over 12 months	HCQ (600 mg) Palbociclib (100 mg QD, 150 mg QD, and 200 mg QD)	Started in June 2023, and will complete in December 2024	Combined
NCT01023737	Advanced solid tumors	Completed	HCQ Vorinostat	1	Safe maximum dose, effectiveness in reducing tumor size	HCQ (400 mg, 600 mg, 800 mg, 1000 mg/day by mouth) Vorinostat (300 mg/day)	Started in November 2009, and its completion date was in January 2023	Combined
NCT02421575	Prostate cancer	Completed	HCQ	1	Autophagy, PSA levels, apoptosis markers, circulating tumor cells	Not defined	Started in July 2012, and its completion date was in February 2016	Monotherapy
NCT05842174	Hepatocellular carcinoma	Not yet recruiting	HCQ Lipiodol	1, 2	Local progression-free survival	Not defined	Started in September 2023, and will complete in August 2028	Combined
NCT05433402	Stage III colon cancer	Withdrawn	ChlorproMAZINE	1, 2	Overall survival, new colon cancer/ polyp’s occurrence time	ChlorproMAZINE (50 mg IV)	Started in July 2022, and its completion date was in September 2022	Combined
NCT02466802	Advanced solid tumors	Completed	Regorafenib Sildenafil Citrate	1	Safety, toxicity, antitumor effects, impact on regorafenib pharmacokinetics	Not defined	Started in July 2015, and its completion date was in January 2019	Combined
NCT01324596	Lymphoma (large B-Cell)	Completed	Bortezomib	3	Overall survival, time to progression, response duration, overall response rates, toxicity	Bortezomib (100 mg by mouth)	Started in April 2011, and its completion date was in June 2015	Monotherapy
NCT06218524	Adult recurrence glioblastoma	Not yet recruiting	Haloperidol Tablets Temozolomide	2	Relief percentage, overall survival, DRD2 expression, haloperidol adverse reactions	Haloperidol tablet (6 mg triple/day by mouth) Temozolomide (150 mg/kg once/day by mouth)	Started in January 2024, and will complete in July 2028	Combined
NCT06076837	Metastatic pancreas cancer	Not yet recruiting	Botensilimab Balstilimab Chloroquine Phosphate Celecoxib	1	Maximum tolerated dose, safety, treatment response, survival rates, biomarker changes	Botensilimab (50 mg IV) Balstilimab (240 mg IV) Chloroquine Phosphate (500 mg) Celecoxib (200 mg twice/day)	Started in December 2023, and will complete in December 2025	Combined
NCT01292408	Breast cancer	Unknown Status	HCQ	2	Changes in hypoxia and autophagy markers	HCQ (400–800 mg)	Started in January 2011, and its completion date was in January 2012	Monotherapy
NCT01430585	Breast cancer	Terminated	PF-04691502 Letrozole	2	Changes in Ki-67, objective response, pharmacokinetic parameters, genetic alterations	Not defined	Started in March 2012, and its completion date was in December 2012	Combined
NCT01697293	Stage IIB-IV breast cancer	Terminated	Cyclophosphamide Doxorubicin hydrochloride	1, 2	Pathologic response rates, safety, biomarker analysis	Cyclophosphamide (600 mg) Doxorubicin hydrochloride (60 mg)	Started in January 2012, and its completion date was in June 2020	Combined
NCT00411788	HER-2+ metastatic breast cancer	Completed	Rapamycin Trastuzumab	2	Objective response rate, cardiac dysfunction incidence, molecular changes	Rapamycin (6 mg/day) Trastuzumab (2–4 mg/kg)	Started in December 2006, and its completion date was in April 2010	Combined
NCT01628913	Pancreatic neuroendocrine tumors	Terminated	BEZ235 Everolimus	2	Progression free survival, overall survival, time to treatment failure	BEZ235 (400 mg twice/day by mouth) Everolimus (10 mg/day by mouth)	Started in October 2012, and its completion date was in September 2019	Combined
NCT01210911	Pancreatic cancer	Completed	Gemcitabine Erlotinib Metformin	2	Objective response rate and toxicity profile	Gemcitabine (1000 mg/m^2^) Erlotinib (100 mg) Metformin (500–1000 mg twice/day)	Started in August 2010, and its completion date was in April 2014	Combined
NCT00786682	Metastatic prostate cancer	Terminated	HCQ Docetaxel	2	Tumor response rate, time to disease progression, overall survival, safety	HCQ (200 mg twice/day) Docetaxel (75 mg/m2 IV)	Started in December 2008, and its completion date was in October 2010	Combined
NCT00003084	Prostate cancer	Completed	Doxorubicin hydrochloride Estramustine phosphate sodium Etoposide	2	PSA-based response rate	Not defined	Started in December 1997, and its completion date was in November 2011	Combined
NCT00657982	Intermediate or high-risk prostate cancer	Unknow Status	RAD001	2	Tumor response rate, PSA failure assessed between 3 to 5 years	RAD001 10 mg/day	Started in April 2008, and its completion date was in March 2010	Monotherapy
NCT01313559	Naive prostate cancer	Terminated	Pasireotide Everolimus	2	Progression-free survival, PSA levels, progression-free survival	Not defined	Started in June 2011, and its completion date was in November 2012	Combined
NCT00574769	Advanced prostate cancer	Completed	RAD001 Docetaxel Bevacizumab	1, 2	Efficacy, dose response	RAD001 (2.5–5 mg/day by mouth) Docetaxel (75 mg/m^2^ every 21 days IV) Bevacizumab (15 mg/kg every 21 days IV)	Started in February 2010, and its completion date was in February 2016	Combined
NCT01433913	Prostate cancer	Completed	Metformin hydrochloride	2	Tumor and serum biomarkers	Not defined	Started in November 2011, and its completion date was in April 2014	Monotherapy
NCT01396200	Multiple myeloma	Completed	HCQ Rapamycin	1	Number of adverse events, the feasibility of treatment	HCQ (600–800 mg/day by mouth) Rapamycin (3 mg, 4 mg, 9 mg, 12 mg/day by mouth)	Started in June 2011, and its completion date was in October 2012	Combined
NCT02631252	Myelogenous leukemia	Terminated	HCQ Mitoxantrone Etoposide	1	Complete response, overall survival, relapse-free survival, pharmacodynamic endpoints	HCQ (600–1400 mg twice/day by mouth) Mitoxantrone (10 mg/m^2^ IVPB in 50 mL NS) Etoposide (100 mg/m^2^ IV)	Started in August 2016, and its completion date was in October 2017	Combined
NCT01227135	CML	Unknown Status	HCQ Imatinib mesylate	2	Safety, efficacy, BCR/ABL levels, drug levels, effects on progenitors	Not defined	Started in March 2010, and its primary completion date was in March 2012	Monotherapy, and Combined
NCT01689987	Relapsed or refractory multiple myeloma	Completed	HCQ Rapamycin Cyclophosphamide Dexamethasone	1	Maximum tolerated dose, myeloma response, progression-free survival	Noy defined	Started in September 2012, and its primary completion date was in April 2016	Combined
NCT01079767	Advanced liver cancer and cirrhosis	Terminated	Temsirolimus	2	3-month disease-control rate, progression-free survival, response rates, overall survival	Not defined	Started in January 2010, and its primary completion date was in December 2010	Monotherapy
NCT01035229	Advanced hepatocellular carcinoma	Completed	Everolimus	3	Overall survival, time to tumor progression, disease control rate, pharmacokinetics	Everolimus (2.5 mg)	Started in April 2010, and its completion date was in October 2013	Monotherapy
NCT00492752	Advanced hepatocellular carcinoma	Completed	Sorafenib	3	Overall survival, time, disease control rate, overall response rate, pharmacokinetics	Sorafenib (400 mg twice/day by mouth)	Started in October 2005, and its completion date was in July 2009	Monotherapy
NCT00522665	Colorectal cancer	Completed	Irinotecan Cetuximab RAD001	1, 2	Maximum tolerated dose, response rates	Irinotecan (125 mg/m^2^ IV) Cetuximab (250 mg/m^2^ IV) Not defined	Started in August 2008, and its completion date was in February 2015	Combined
NCT01628913	Colorectal cancer	Terminated	MK-2206	2	Overall objective response rate, overall survival, safety, tolerability, adverse event profiles	MK-2206 (200 mg by mouth)	Started in August 2010, and its completion date was in August 2011	Monotherapy
NCT01941953	Refractory colorectal cancer	Completed	Metformin 5-FU	2	Disease control rate, progression-free survival, overall survival	Metformin (850 mg) 5-FU (425 mg/m2)	Started in November 2012, and its completion date was in March 2015	Combined
NCT01460979	Ovarian carcinoma or advanced endometrial carcinoma	Completed	Temsirolimus	2	Progression-free survival, safety, stable disease rates, overall survival	Temsirolimus (25 mg)	Started in October 2010, and its completion date was in November 2015	Monotherapy
NCT01031381	Ovarian, peritoneal, and fallopian tube cancer	Completed	RAD001 Bevacizumab	2	Progression-free survival, treatment response	RAD001 (10 mg once/day by mouth) Not defined	Started in September 2010, and its completion date was in December 2014	Combined

## Abbreviations

ATG, autophagy-related genes; TNBC, triple-negative breast cancer; LC3, the tubule-linked protein 1-light chain 3; AMPK, AMP-activated kinase; mTOR, mechanistic target of rapamycin kinase; 3-MA, 3-methyladenine; spautin-1, specific and potent autophagy inhibitor-1; CQ, chloroquine; HCQ, hydroxychloroquin; PI3K, phosphoinositide 3-kinase; CPT, camptothecin; PSD, Pulsatilla saponin D; Baf A1, bafilomycin A1; RNAi, RNA interference; ERK, extracellular signal-regulated kinase; MAPK, mitogen-activated protein kinase; AZA, azacitidine; CML, chronic myeloid leukemia; IM, imatinib mesylate; siRNA, small interfering RNA; MM, multiple myeloma; DAB, dabrafenib; TRA, trametinib; ROS, reactive oxygen species; 5-FU, 5-fluorouracil; DTCs, disseminated tumor cells TCM, traditional chinese medicine; TSN, toosendanin; ULK1, Unc-51-like kinase 1.
